# Five QTL hotspots for yield in short rotation coppice bioenergy poplar: The Poplar Biomass Loci

**DOI:** 10.1186/1471-2229-9-23

**Published:** 2009-02-26

**Authors:** Anne M Rae, Nathaniel Robert Street, Kathryn Megan Robinson, Nicole Harris, Gail Taylor

**Affiliations:** 1 School of Biological Sciences, University of Southampton, Bassett Crescent East, Southampton, SO16 7PX, UK

## Abstract

**Background:**

Concern over land use for non-food bioenergy crops requires breeding programmes that focus on producing biomass on the minimum amount of land that is economically-viable. To achieve this, the maximum potential yield per hectare is a key target for improvement. For long lived tree species, such as poplar, this requires an understanding of the traits that contribute to biomass production and their genetic control. An important aspect of this for long lived plants is an understanding of genetic interactions at different developmental stages, i.e. how genes or genetic regions impact on yield over time.

**Results:**

QTL mapping identified regions of genetic control for biomass yield. We mapped consistent QTL across multiple coppice cycles and identified five robust QTL hotspots on linkage groups III, IV, X, XIV and XIX, calling these 'Poplar Biomass Loci' (PBL 1–5). In total 20% of the variation in final harvest biomass yield was explained by mapped QTL. We also investigated the genetic correlations between yield related traits to identify 'early diagnostic' indicators of yield showing that early biomass was a reasonable predictor of coppice yield and that leaf size, cell number and stem and sylleptic branch number were also valuable traits.

**Conclusion:**

These findings provide insight into the genetic control of biomass production and correlation to 'early diagnostic' traits determining yield in poplar SRC for bioenergy. QTL hotspots serve as useful targets for directed breeding for improved biomass productivity that may also be relevant across additional poplar hybrids.

## Background

There is currently a new wave of interest in the use of biomass as a renewable fuel source, both for heat and electricity production as well as for liquid transport fuels such as bioethanol, from biochemical fermentation or bio-oil from thermo-chemical conversion. This is particularly true for second generation lignocellulosic crops that are unlikely to compete with food crops on agricultural land. Irrespective of end use, yields of current second generation crops remain a limiting factor to commercial establishment, since they are largely unimproved, with current commercial yields falling far short of both theoretical and experimental yield maxima [1]. Fast-growing tree species such as poplar (*Populus*) and willow (*Salix*) that can be grown as short-rotation coppice (SRC) represent one of the most appealing sources of renewable biomass feedstock [2] and have significant yield potential [3]. SRC crops are easy to establish, provide a fuel source that is multi-functional, as well as offering secondary benefits such as low nutrient input, good energy balance, bioremediation abilities, and increased biodiversity [4,5]. However, to date, breeding efforts and scientific studies have concentrated on single-stem growth of poplars and there is a need to identify traits and genomic loci as targets for the development of improved SRC biomass-yielding genotypes.

Woody biomass yield is a highly complex trait as it represents the integrated and combined result of many other complex traits, each themselves under polygenic control. In order to inform breeding for biomass improvement, it is therefore important to understand the traits that contribute to biomass production and to locate the loci involved in the control of those trait components before then moving on to identify the specific desirable allelic variants. We have previously performed a multivariate analysis of phenotypic traits and modelled their contribution to biomass production in the population used here [6].

In a long-lived species such as poplar, it is also essential to understand how biomass production changes with maturity (or in the case of SRC, the individual stools and the entire stand) with understanding being required at the genetic and physiological/morphological level. A number of studies have reported QTL in the population used for this study at a single time point, for single stem plants usually during early phases of growth [7,8]. However, several studies report different QTL at different time points or plant age [9-11]. Interpretation of such results can be ambiguous as to whether these are true differential effects with time, or statistical issues resulting from factors such as low sample size or replication. There is currently no available QTL information on traits related to coppice growth.

In the present study, QTL mapping and genetic correlations were used to examine interactions and temporal relationships between biomass-associated traits and to identify key loci controlling those traits in SRC.

## Results

### Trait variation

There was nearly a 30 fold variation in biomass yield for the final biomiass (CC2-4) harvest with genotypic mean values ranging from 0.58 Kg to 16.3 Kg. The degree of variation for the CC1-1 harvest was far greater with nearly a 100 fold range in yield and the rank order of genotypes differed between the two. Biomass at both CC1-1 and CC2-4 was skewed towards lower values. The largest variation in any trait for the CC2-4 data was seen for total basal diameter where there was >150 fold difference between the genotypic mean min and max values. Height showed the least variation with a minimum value of 0.98 m and a maximum of 6.99 m. These trends were similar for the CC1-1 data. The number of coppice stems varied from 1 to 24 and there was considerable variance in the consistency of the diameter of each stem, with some genotypes having a clearly identified leader and others having many stems of more uniform size.

### Physiological trait correlations

**Figure 1:**
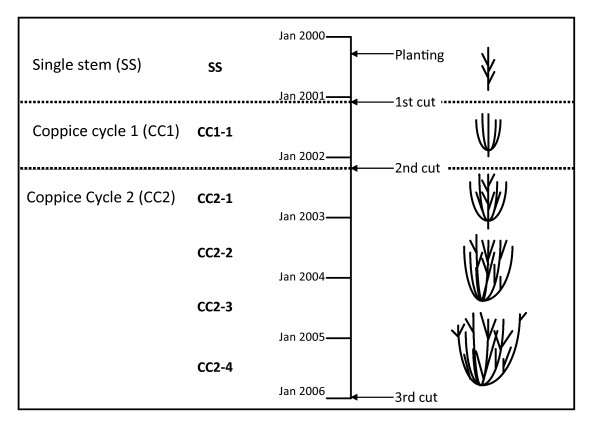
**Experimental overview**. Diagramatic representation of the planting and copice cycle timeline.

We have previously reported heritability values and a multivariate analysis of trait contributions to biomass yield for coppice cycle 1 (CC1. See Table 1 and [6]. See [12] for details of Leaf Plastochron Index.). The data in [6] are represented here as SS (Single Stem equivalent to days after planting, DAP) and CC1 (Coppice Cycle 1 equivalent to days after coppice, DAC). Here we present the results of QTL analysis for the data presented in [6] with the addition of a final biomass harvest following an extra coppice cycle of 4 years (CC2). An overview of the coppice cycle is shown in Figure 1.

**Table 1:** Details of traits for which QTL were mapped

**Trait**	**Details**	**Description**	**Date**	**Units/details**
SYL	SS	SS number of branches	Dec 2000	
DIAM	SS	SS Diameter	Dec 2000	mm
HT-1	SS	SS Height	Dec 2000	m
HT-2	CC1-1	Height of leader	Jun 2001	m
Leaf-prod	CC1-1	Leaf production rate	Jun 2001	Leaves day^-1^
No-leaves	CC1-1	Leaf number on leader	Jun 2001	
STM-No	CC1-1	Number of coppice shoots	Jun 2001	
Pet_length	CC1-1	Petiole length	Jun 2001	mm
PI-1	CC1-1	Plastochron Index^§^	Jun 2001	
STM_ext	CC1-1	Stem extension increment of leader	Jun 2001	mm day^-1^
C13	CC1-1	Carbon isotope discrimination	Jul 2001	‰
Leaf_area-1	CC1-1	Area of largest mature leaf on leader	Jul 2001	cm^2^
Leaf-ext	CC1-1	Extension rate of leaf Ln+1^§^	Jul 2001	mm day^-1^
Leaf-prod-1	CC1-1	Leaf production rate	Jul 2001	Leaves day^-1^
No_leaves-1	CC1-1	Leaf number on leader	Jul 2001	
PI	CC1-1	Plastochron Index^§^	Jul 2001	
SLA	CC1-1	Specific Leaf Area (largest leaf on leader)	Jul 2001	mm^2 ^g^-1^
Cell-Area	CC1-1	Cell area of largest mature leaf	Aug 2001	*μ*m^-2^
Cell-No	CC1-1	Calculated cell number	Aug 2001	leaf area/cell area
Leaf_area-2	CC1-1	Area of largest mature leaf on leader	Aug 2001	cm^2^
Budburst	CC2-1	Week of leaf flush (29^th ^March to 9^th ^May)	2002	
HT-3	CC1-1	Height of leader	Feb 2002	m
STM-No-1	CC1-1	Number of coppice shoots	Feb 2002	
Biomass	CC1-1	Calculated dry biomass yield*	Feb 2002	kg
B-DIAM	CC2-1	Total basal diameter of all coppice shoots	Feb 2002	mm
B-AREA	CC2-1	Total basal diameter of all coppice shoots	Feb 2002	mm^2^
STM_Vol	CC2-1	Stem volume index of leader*	Feb 2002	
HT-4	CC2-4	Height of leader	Jan 2006	m
B-DIAM-1	CC2-4	Total basal diameter of coppice shoots	Jan 2006	mm
B-AREA-1	CC2-4	Total basal area of coppice shoots	Jan 2006	mm^2^
STM-No-2	CC2-4	Number of coppice shoots	Jan 2006	
Biomass-1	CC2-4	Calculated dry biomass yield*	Jan 2006	Kg

Details of traits for which QTL were mapped. SS refers to Single Stem. CC refers to Coppice Cycle where the first number indicates the cycle number and the second number the year within that cycle. ^§^Refer to [12] for details. *Refer to [6] for details.

**Figure 2:**
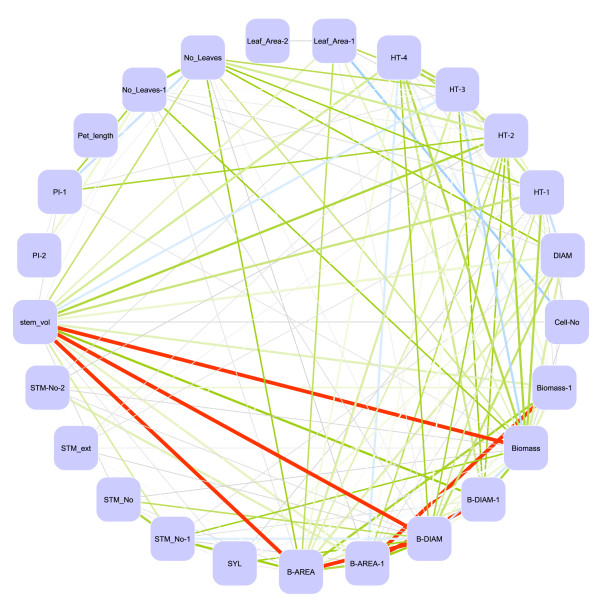
**Penotypic trait correlations**. Visualisation of Spearman's rank correlations between traits. Non-significant correlations and those < 0.5 are not shown. Line width and colour represent correlation values between traits. All correlations below 0.7 are in grey, > 0.7 in green, > 0.8 in blue and > 0.9 in red. Line widths are increased from 0.6 to 0.9 in 0.1 increments for emphasis. Thick red lines show the strongest correations between traits.

Strong phenotypic correlations were found between biomass, height and diameter traits, particularly within the same year of measurement (Figure 2). Biomass accumulation in the first and subsequent years remained a reasonable predictor of biomass yield through to CC2-4 (Biomass 1), suggesting that early screening for elite genotypes would be a reliable indicator of sustained productivity. Weaker yet interesting correlations were also found. For example, cell number has been seen to be a more important determinant of biomass, height and leaf area than is cell area [6]. As well as individual leaf area showing significant correlations to biomass traits, leaf number was also important (both having positive correlations). The number of stems was consistently an important determinant of biomass accumulation within this experimental design.

Genetic correlations (Table 2, Additional file 1) showed a similar pattern with strongest correlations being between biomass, height and stem diameter within years. However, traits scored in the first and second year showed low genetic correlations to those of the final harvest measurements (e.g. r = 0.187 +/- 0.05 between biomass in CC1-1 and biomass in CC2-4). Genetic correlations also showed leaf area to be moderately correlated to height1, diameter and sylleptic branch number, stem number, height3 and stem number 2 (Additional file 1). Two-way ANOVA (data not shown) showed highly significant time, and genotype × time interactions for height, diameter, number of stems and biomass (p < 0.001 in all cases).

**Table 2:** Genetic correlation between traits

	Biomass	Biomass-1	DIAM	B-DIAM	B-DIAM-1	HT-1	HT-2	HT-3
Biomass-1	0.19							
DIAM	0.59	0.05						
B-DIAM	0.62	0.22	0.37					
B-DIAM-1	0.12	0.63	0.07	0.11				
HT-1	0.54	0.08	0.77	0.35	0.05			
HT-2	0.37	-0.04	0.38	0.25	0.07	0.34		
HT-3	0.36	0.02	0.35	0.3	0.02	0.36	0.3	
HT-4	0.09	0.38	0.1	0.08	0.45	0.1	-0.02	0.03

Genetic correlation between selected biomass-related traits. Trait name abbreviations are given in Table 1.

See Additional file 1 for the full correlation matrix of all traits and for standard error values. Genetic correlations between traits were calculated from the variance-covariance matrices obtained from a multivariate Aanlysis Of Variance anlaysis.

### QTL mapping

**Figure 3:**
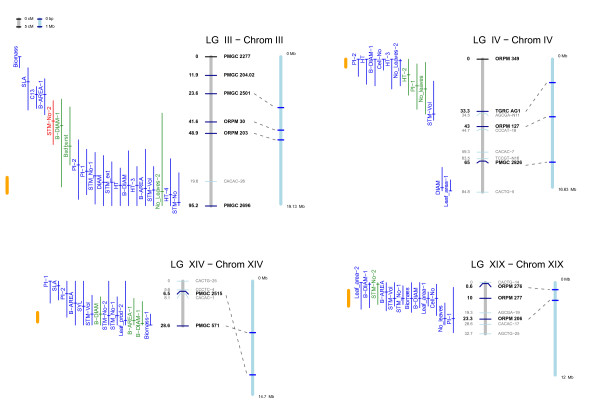
**QTL for biomass-associated physiological traits**. QTL are plotted +\- confidence intervals defined as an F2 drop off with peak F score location being marked with a short horizontal mark. QTL explaining < 5% variance are plotted in blue, those explaining 5–8% in green and > 8% in red. The genetic linkage group for Family 331 is plotted on the left in grey with cM locations of markers shown to the left and marker names to the right. SSR markers are plotted as dark blue lines with black text, AFLP markers in light blue with grey text. The chromosome is plotted on the right in blue with SSR locations marked as blue horizontal lines. Dotted lines connect SSRs between the linkage map and chromosome where possible. Orange lines represent regions over-represented with co-locating QTL identified using a sliding window approach.

In total 207 QTL were mapped (Table 3, Additional file 2) with an average of 5.6 QTL mapped per phenotypic trait and 9.4 QTL per linkage group (LG). The number of QTL mapped per LG varied greatly (standard deviation of 6.1) with apparent QTL 'hot spots' on LGs III and X (22 and 21 QTL respectively) with other LGs having very few mapped QTL. There were additional clusters of QTL on LGs IV, VIIIa, IX, XIV, and XIX (Figure 3) and LG II, XIII and XVIII narrowly missed having hotspots declared using the sliding window criteria. QTL mapped explained a mean 4% of phenotypic trait variance (% Vp) with a maximum of 9.8% for any single QTL (height4; Additional file 2) and a maximum 41.5 total % Vp for a single trait (Plastochron index; Table 4, Additional file 2). Mapped QTL explained a mean 3% Vp in biomass yield across the two biomass harvests.

**Table 3:** Mapped QTL explaining > 5% Vp for biomass-related traits

**Trait**	**LG**	**CI**	**Paternal**	**Maternal**	**% Vp**	**PBL**
Biomass-1	VIIIa	0–9	-0.66	-0.55	6.7	2
Biomass	IX	0–9	-0.39	-0.45	5	
Biomass	X	31–56	-0.36	-0.73	5.2	3
B-DIAM	I	25–51	-7.23	-3.69	5.3	
B-DIAM-1	III	27–83	13.98	10.20	5.8	
B-DIAM	Va	31–61	-4.41	-8.13	5.2	
B-DIAM	IX	0–9	-5.37	-5.2	5	
B-DIAM-1	X	0–15	23.52	7.05	6.2	3
B-DIAM	X	31–57	1.35	-8.40	5.2	3
DIAM	X	37–57	0.93	-13.98	7.5	3
B-DIAM	XIV	10–28	2.04	-7.05	5.5	4
B-DIAM-1	XIV	13–28	6.60	-20.96	5.3	
B-DIAM-1	XVIII	27–47	-24.61	9.96	8.1	
						
HT-4	II	20–39	0.41	0.0022	9.8	1
HT-2	II	22–40	0.0047	-0.013	5.3	1
HT-2	IV	0–23	-0.027	-0.032	6.6	
HT-4	X	26–56	0.11	-0.38	8.7	3
HT-3	X	32–62	-0.022	-0.16	5.2	3
HT-1	XIII	45–60	0.030	-0.071	7.2	

Mapped QTL explaining > 5% Vp for biomass-related traits. Trait name abbreviations are given in Table 1. Confidence Intervals are the cM distances where the peak F score drops by 2 units. % Vp – percentage phenotypic trait variance explained. PBL refers to the inclusion of a QTL in a Poplar Biomass Loci as described in the main text.

**Table 4:** Total phenotypic variance explained by mapped QTL

**Trait**	**Total % Vp**
B-DIAM	41.5
PI-2	40.25
STM_No-2	38.13
B-AREA-1	36.8
B-DIAM-1	36.6
No_Leaves-2	33.5
HT-4	33
SYL	32.4
PI-1	31.5
Budburst	30.8
B-AREA	29.7
SLA	26.57
STM-Vol	25.7
STM-No	25.5
No_Leaves	24.5
HT-2	24.4
HT-3	23.9
STM_No-1	23.9
C13	21.6
Biomass-1	21.4
Cell-No	21.1
Leaf_area-2	20.9
HT-1	20.1
DIAM	18.5
Leaf_prod-2	18.46
Leaf_area-1	18.2
Biomass	17.7
Leaf-ext	11.6
STM_ext	10.7
Cell-Area	6.6
Leaf_prod-1	2.8
Pet_length	1.8

Cumulative % Vp (percentage phenotypic trait variance explained) for all traits used for QTL mapping. Trait name abbreviations are given in Table 1.

#### Biomass, height, stem volume and diameter

A number of QTL for direct biomass related traits (i.e. height and diameter) were mapped consistently in multiple years of growth with consistent % Vp and direction of maternal and paternal effects (Table 3). In some cases, QTL for diameter and height both co-located with QTL for biomass and in other cases only co-location of biomass to height or diameter was observed, allowing inference to be drawn about the underlying architecture of these QTL.

**Figure 4:**
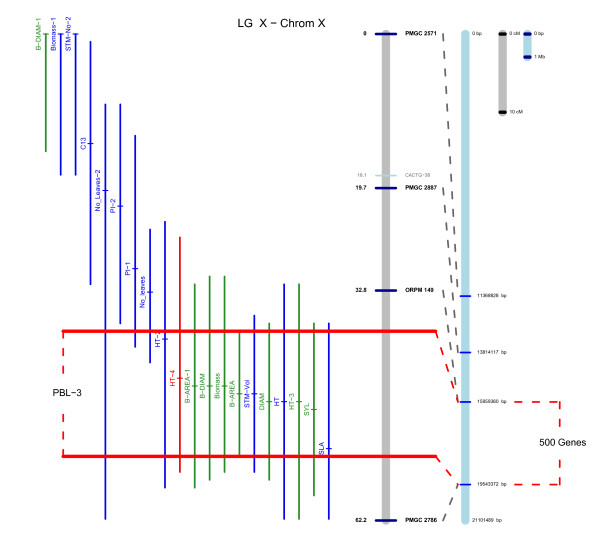
**QTL located in Poplar Biomass Loci 1 on Linkage Group X**. QTL are plotted +\- confidence intervals defined as an F2 drop off with peak F score location being marked with a short horizontal mark. QTL explaining < 5% variance are plotted in blue, those explaining 5–8% in green and > 8% in red. The genetic linkage group for Family 331 is plotted on the left in grey with cM locations of markers shown to the left and marker names to the right. SSR markers are plotted as dark blue lines and black text, AFLP markers in light blue with grey text. The chromosome is plotted on the right in blue with SSR locations marked as blue horizontal lines with bp positions to the right. Dotted lines connect SSRs between the linkage map and chromosome where possible.

Consistent QTL for diameter traits (basal diameter, basal area, and diameter of leader) were located on LGs I, III, X, XIII, XIV, XV, and XIX. There was a general tendency towards the paternal parent (*P. deltoides*) contributing a positive effect and the maternal parent (*P. trichocarpa*) a negative one. Those QTL on LGs X and XIV co-located to QTL for biomass yield. In the case of LG X, the diameter QTL co-located to QTL for height (Figure 4). In contrast, the biomass QTL on LG XIV was influenced only by diameter. Other QTL appeared to be specific to certain years. For example QTL specific to CC2-1 were mapped on LGs V, IX, and XVII and to CC2-4 on XVIII.

Height QTL were consistently mapped to LGs II, III, IV, and X. The QTL on LGs II and X explained relatively high % Vp compared to other QTL within this study (9.8 and 8.7% CC2-4) and in both cases, explained increasing % Vp over time. However, % Vp was reduced when early height measurements were used as covariates for mapping QTL of later height measurements within the same coppice cycle.

#### Stem number and sylleptic branches

For the three measures of stem number, mapped QTL explained between 23 and 38% Vp. A significant and positive correlation between stem number and biomass yield as well as height and diameter was found. Only one QTL on LG XIV was mapped consistently for these traits across all years. For SS measurements (the only year in which sylleptic branches were examined) there were a number of cases where QTL for sylleptics co-located to QTL for stem number, suggesting that these two traits may be under common genetic control. Co-locating QTL can be seen on LGs III, V, VIII, XIII, and XIV. For CC2-4 data, mapped QTL explained a total of 38% Vp for number of stems with two QTL explaining > 9% Vp (LG III and XVIII).

#### Leaf area and cell traits

QTL explaining between 18 and 21% Vp in leaf area were mapped with an individual maximum % Vp of 7.5 for a QTL on LG VI. No co-locations between QTL for cell area and leaf area were found but co-locating QTL for leaf area and cell number were found on LGs IX, XVII, and XIX. In total QTL explaining 21% Vp for cell number were mapped. In contrast, very few QTL for cell area were mapped, explaining a total 8.9% Vp. No co-location of QTL for leaf extension rate, cell number and/or leaf area was seen. Co-locations between leaf area related and biomass related QTL were found on LGs II, III, IV, VII, IX, XIX. There was a co-locating QTL for cell number and specific leaf area on LG II.

#### Canopy traits

QTL explaining 30.8% Vp for spring bud flush were mapped. The % Vp values reported here for bud flush are considerably smaller than those reported by [7] but as [13] point out, the values in [7] are likely over-estimates (due to the Beavis effect, see [14] for a mathematical explanation) and the values reported here are more reliable due to the higher number of genotypes used for our QTL mapping. However, our values are still likely over-estimates [15].

A number of co-locations were found between leaf number, leaf production and Plastochron Index (PI), however these are to be expected as the traits are highly related measures. In such a case, co-location can be considered to indicate that the QTL are reliable [16-20]. Co-location between biomass yield and PI or leaf number was found on LGs VII, XIX, and X. Co-location between bud burst and biomass was found on LG VIIIa.

### Candidate loci for biomass yield in poplar

We identified clusters of co-locating QTL on LGs II, VIIIa, X, XIV, and XIX that we have termed Poplar Biomass Loci (PBL; Figure 3, 4). All but the cluster on LG II and VIIIa were also identified as hotspots for co-location using the sliding window over-representation approach. In order to be classified as a PBL we used the arbitrary set of criteria that QTL clusters had to have at least one QTL explaining > 5% Vp, contain a QTL for biomass yield and have consistent maternal and paternal effects. Of these, PBL-3 (Figure 4) on LG X is perhaps the most interesting and we therefore examined this PBL in greater detail. PBL-3 contains co-locating QTL for height and diameter in multiple years in addition to QTL for biomass (CC1-2), stem volume, sylleptic branch number, and specific leaf area with the majority of QTL explaining > 5% Vp. Although other loci could also be termed PBL, we feel that these are currently the most important and well defined.

### Genes within QTL regions

The release of the poplar genome sequence [21] and the use of sequence based markers (i.e. SSRs) for map construction allowed us to identify gene models between flanking SSR markers on the genetic map for Family 331 (Figure 4). On average, there were ~600 gene models between flanking SSR markers (data not shown).

## Discussion

Here we report results of QTL mapping in a partially inbred F_2 _population of out-breeding poplar, Family 331, grown as SRC. In general we mapped QTL explaining a relatively large % Vp for numerous traits associated with biomass yield (Figures 3 and 4, Table 2 and Additional file 2 see [6] for an in-depth multivariate analysis of SS and CC1 data), typically with few QTL explaining the largest percentage of trait variation (Additional file 1). Previous studies conducted in the USA on this, and a related back-cross population, grown as both single stem [7,8,22-25] and SRC [26] and at three sites across Europe [27] reported similar results. This is the first study in *Populus *to report the genetic control of traits important to growth as short rotation coppice. Biomass at both CC1-1 and CC2-4 was skewed towards lower values, a result also reported in [26]. This is likely the outcome of inbreeding depression and the resultant high genetic load associated with an inbred F_2 _population derived from out-breeding species [28]. The number of coppice shoots varied from 1 to 24 and there was considerable variance in the consistency of the diameter of each shoot, with some genotypes having a clearly identified leader and others having many shoots of more uniform size. This trait is likely to be tightly related to the strength of apical dominance. Stem number may also be affected by environmental factors such as planting density. In this study, the trees were grown 1 m apart, towards the lower planting density for commercial field trials, so the amount of variation displayed for this trait may differ for more densely planted trials. Sylleptic branching is also a function of the strength of apical dominance and a significant correlation was found between sylleptic branch number and stem number 1 although no co-locating QTL for the two traits were observed. The relatively low genetic correlations of stem traits between CC1-1 and CC2-4 and phenotypic biomass traits between years suggest differential genetic control of these traits with tree age, although correlations between years for genotypes at the phenotypic extremes were more consistent. The decreasing genetic correlations between height and biomass between years are in agreement with the theory that biomass produced early on is largely related to plant height, but as the tree ages, increased biomass appears to be due to increased girth [24].

A number of QTL were mapped consistently across different years, in some cases being present for all datasets recorded. From a breeding perspective these may represent the most important targets for directed breeding, as QTL mapped consistently across years and multiple environments represent those that are least likely to be affected by GxE interactions. In the current experiment, the population was grown for two coppice cycles and therefore QTL mapped for the CC2-4 data and common to other years are good targets for genotype improvement in this genetic cross.

### QTL co-location: Five key Poplar Biomass Loci

At multiple positions we identified clusters of QTL, often for correlated and allometrically linked traits such as height or diameter and biomass and leaf area. In particular, we found clusters of QTL on LGs II, III, IVa, VIIIa, X, XIV, XVIII, and XIX, with the clusters on II and X being particularly interesting. In the case of LG X we were previously able to show that this is equivalent to LG J from previous mapping work presented in papers by Bradshaw et al. [27], where QTL for biomass were also identified. In all work on this population in both the USA [29], in this study, and in [27] LG X has been universally mapped in relation to biomass yield suggesting that this is a highly robust QTL with consistent effect across environments and growth practices. LG XIX is the same as LG O in the work by Bradshaw and here again, QTL for similar traits (stem number) were identified.

From both the biological and breeding perspective it is of interest to examine positions with co-locating QTL while simultaneously considering phenotypic correlation values (or VIP scores in respect to [6]) and genetic correlations. We identified many genomic loci with co-locating QTL. In some cases these appear to be loci affecting the control of many traits and in others, they are specific to a particular trait and its most allometrically related or closely correlated traits (e.g. height and biomass).

LG II (PBL-1) is an example of co-locating QTL that appear largely specific to height. QTL for height were mapped in all cases where height was recorded for CC data. This cluster includes QTL for CC2-4 biomass, stem extension rate as well as a QTL for leaf extension rate, which is close enough to suggest co-location. There are various different interpretations of what the underlying causative mechanism of this QTL may be: As the locus appears to affect both stem and leaf extension rate, it is possible that the rate of cell division or expansion (or both) is rate-limiting; however, an alternative hypothesis is that the increased extension rate of leaves results in more rapid development from sink to source. Leaf area (particularly on the terminal shoot) is more tightly correlated to height than diameter [22,30] and so more rapidly maturing leaves would lead to a more rapid increase in height extension.

LG VIIIa contains co-locating QTL for sylleptic number and the number of coppice stems in addition to bud flush and height (height4). Speculating as to a causative mechanistic link between all of these traits is difficult and trait and genetic correlations do not suggest a link between them. It is therefore possible that this cluster of QTL represents more than one gene but that either our mapping resolution is insufficient to distinguish the two adequately or they are in linkage and are being co-inherited as a single locus.

LG X (PBL-3; Figure 4) contains multiple co-locating QTL for both height and diameter, with many explaining high % Vp within the context of this experiment. It is possible that this represents the location of a gene affecting the activity of the cambial meristem region and we are currently examining evidence from literature sources concerning gene expression, mutational/over-expression studies and genes of known biological function in xylem formation and cambial activity to identify likely candidate genes. LG XIV (PBL-4; Figure 3) represents a QTL cluster more specific to diameter. The presence of QTL for stem number in CC1-1 and CC2-4 suggest that the causative mechanism for this QTL may well be an increase in diameter along with increased stem number. This relationship is not unidirectional as there was clear segregation in the F_2 _for both traits with differences in the genotypic rank order for both traits.

LG XIX (PBL-5; Figure 3) contains an interesting cluster of co-locating QTL for basal area, stem number, leaf area and cell number and we are particularly interested in the observed correlations between cell number on the abaxial leaf epidermal surface with both leaf area and biomass traits, a result also found for various willow genotypes [31]. The chromosomal region between the flanking SSR markers currently contains only 76 gene models, and none of those with informative annotations can easily be ascribed a role in any of these traits.

Although each trait within a QTL hotspot might only contribute a small positive effect on biomass yield, the co-location of multiple traits indicates a common genetic control mechanism (i.e. pleiotropy) suggesting that selection for the beneficial allele at that locus will result in a cumulative increase in biomass due to the integrative effects of the individually small, positive contributions of the various traits. Where such hot spots contain QTL for traits that are not tightly allometrically linked, it is likely that they represent *trans *acting QTL (most likely transcription factors) where the effect of alterations in regulation or structural characteristics would be expected to have smaller-scale effects but potentially on many traits. In contrast, *cis *acting QTL are more likely to have large-scale effects but on a single trait or a far more limited set of highly related traits. Pleiotropic loci may, however, result from tight linkage between genes within the same chromosomal region. Examination of modes of action may help draw inferences but further dissection of such loci is required. In some cases it may be implausible or at least highly unlikely that two allometrically related traits are influenced by the same gene. Careful consideration of such possibilities is especially important if results from inter-specific crosses are to be used to direct breeding in other related species; the same allometric relationship, or link between allometry and genetic control, may not exist in alternative genetic backgrounds that have been exposed to different selection pressures and certain QTL may result from unique epistatic interactions created within the inter-specific cross. However it is interesting to note that in another poplar F_1 _cross grown at two contrasting sites, LG X and XIX were also identified as linkage groups where QTL for biomass related traits were apparent [32].

### Identifying genes underlying QTL

A major challenge in bridging the gap between QTL and the underlying, causative DNA polymorphism is the lack of resolution associated with QTL mapping, especially in forest tree species where multi-generation inbred populations cannot be developed. It is for this reason that it has recently been proposed that QTL mapping be used as a pre-screening method to direct subsequent fine mapping in a natural population (i.e. association mapping), where historic recombination is utilised to offer far greater mapping resolution – in the case of poplar down to the individual gene level [33]. Even such an integrated approach is not simple: in the current study we found a mean of just under 600 genes within our QTL hotspots. As linkage disequilibrium breaks down very rapidly in natural populations of poplar [33] this would require developing SNP markers for all of those genes. Even then, the assumption is that linkage exists to the causative polymorphism within the coding region of the gene, which may not be the case where the causative polymorphism lies within the upstream or downstream regions of a gene. Certain factors may improve this situation. Street and co-workers [34] proposed that candidate genes can be selected by identifying genes with differential expression between genotypes at the extremes of a phenotypic trait distribution. Here, the assumption is that these genotypes are fixed for the alleles contributing positive and negative effects on the phenotype, and additionally that gene expression plays an important role in determining phenotype. Alternatively the list of genes within a QTL hotspot (or individual QTL CI) can be examined and a 'short list' determined based on available annotation information. Although we examined the functional annotation of genes in identified QTL hotspots, with many hundreds of genes exisiting in each, this is not a viable exercise. This is especially true considering the complexity of, and number of contributing traits to, biomass production. We are therefore undertaking work to examine differences in gene expression between the population extremes for biomass yield.

## Conclusion

We have identified QTL mapped consistently across multiple coppice cycles in poplar grown as SRC and have defined the five most robust QTL clusters as Poplar Biomass Loci 1–5. In total, 20% of the variation in final harvest biomass yield was explained by mapped QTL. These findings both inform our understanding of the complex and integrative process of biomass yield production as well as providing a short list of the most suitable genomic loci that should be considered in targeted breeding programs using this genetic cross.

## Methods

Plant pedigree

The inbred F_2 _population was created from a cross between a female *P. trichocarpa *(clone 93–968 from western Washington, USA) and a male *P. deltoides *(clone ILL-129 from central Illinois, USA). Two siblings, 53–242 (female) and 53–246 (male), from the resulting F_1 _family (Family 53; [35]) were crossed in 1988 to form an F_2 _family of 90 genotypes and again in 1990 to obtain an additional 320 genotypes (Family 331; [26,29]. This pedigree was imported into the UK in 1999.

A replicated field trial (n = 3 planted in a randomised block design of spacing 1 × 1 m) was conducted in the UK at the Forestry Commission field site, Headley, U.K. (51°07' N, 0°50'] W). The trial was established from 25 cm un-rooted hardwood cuttings of 93–968, ILL-129, the two F_1 _parents and 300 F_2 _genotypes.

Cuttings were derived from a stool bed at the University of Washington, Seattle, USA. Planting details have been described previously [6]. Cuttings were planted during spring 2000.

The Single Stem (SS) plants were cut back to initiate the first Coppice Cycle (CC1) on 11^th ^January 2001. CC1 was harvested after one year of growth (CC1-1: year 1 of CC1) in winter 2001–2 to initiate a second coppice cycle (CC2). CC2 was harvested in winter 2005–6 (CC2-4: year 4 of CC2). Only two of the three replicate blocks were measured for CC2 and so all final harvest measurements and QTL mapping are based on n = 2 reps. The date of measurement and replication for all traits is indicated in Table 1.

### Traits measured

Details of all traits measured prior to 2006 can be found in [6]. In 2006, end of coppice cycle biomass, total basal diameter, the number of coppice stems and the height of the leader (the largest coppice stem) were recorded as detailed for previous growing seasons in [6].

### Statistical analysis

Micro-environmental effects were minimised using Papadakis spatial correction [36], based on a 7 × 3 grid on individual data implemented as a set of custom-written functions (pers. comm. Bastien C, INRA Oreans, France) in R [37]. ANOVA were carried out for all traits in R using the 'aov' function with the following model:

Y_ij _= *μ *+ B_i _+ G_j _+ *ε*_ij_

where *μ *is the general mean, B_i _is the effect of block, considered as fixed, and G_j _is the effect of genotype j, considered as random.

Phenotypic correlations between traits were tested for using Spearman's Rank correlation and hierarchical clustering was then performed on the trait correlation matrix after removal of insignificant correlations and traits with no significant correlations to any other traits using an R script.

Genetic correlations between traits (*r*_g_) were calculated from the variance-covariance matrices obtained from the multivariate ANOVA as *r*_g _= Cov_*G*_(*x*, *y*)/√[*σ*^2^_*G*_(*x*) *σ*^2^_*G*_(*y*)], where Cov_*G*_(*x*, *y*) is genetic covariance between traits *x *and *y*, estimated by equating the mean co-products with their expected values according to the Henderson III procedure [38].

In addition, traits that were measured at different time points were analysed for genotype by age interaction by carrying out a two way ANOVA in R using the 'aov' function with the following model:

Y_ij _= *μ *+ A_i _+ G_j _+ *ε*_ij_

where *μ *is the general mean, A_i _is the effect of plant age is considered as fixed, and G_j _is the effect of genotype j considered as random.

### QTL mapping

All genotypes used for QTL mapping were full-sib progeny (referred to here as the F_2 _generation) of Family 331. QTL were mapped using the freely available web-based program QTLExpress [39]. The out-breeding module of the program was used. Permutation testing implemented in QTLExpress was used to establish the critical F value for declaring a QTL present (1000 permutation, see [40]). QTL confidence intervals (CIs) were calculated using a two F drop-off (the cM distance taken for the peak F value to drop by two). The genetic linkage map used was produced by Tuskan et al. (pers. comm.) and consisted of 91 SSR markers genotyped on 350 of the full-sib progeny and 92 fully informative Amplified Fragment Length Polymorphisms (AFLPs) genotyped on 165 genotypes of the progeny. The resulting genetic map consists of 22 Linkage Groups. Where more than one LG has been assigned to a chromosome, they are numbered with the LG number and a letter, with letter order indicating the order of LGs along the chromosome. SSR primer sequences [41] were located on the genome sequence to align the genetic and physical maps and to provide correct orientation of linkage groups (i.e. 3' to 5'). The location information of SSR markers was used to generate gene lists of all genes between flanking SSR markers of a subset of QTL.

QTL figures were produced using a custom-written R package developed by ourselves and available on request. This package implements a permutation test and sliding window approach to identify regions of the genetic map over-represented with co-locating QTL [42]. For each permutation, QTL are randomly shuffled across the genome and a sliding window of 5 cM is then used to count the number of QTL in each window region. The window was advanced in 1 cM steps across the entire genetic map and the maximum number of QTL in a window region was recorded per permutation. The permutation maximum count results were then sorted and used to determine the critical value at a *α*0.05 significance level (the 950^th ^value for 1000 permutations). The sliding window was then applied to the original QTL data to identify regions with more than the critical number of co-locating QTL. The critical number for our data was five (1000 permutations). Identified hotspots should be viewed with caution where traits have been measured repeatedly or where derived traits are calculated (such as stem volume) as these can artificially inflate the chances of co-location occurring.

### Linking the physical sequence to the genetic map

In order to extract lists of genes within QTL regions, the amplified products of primer sequences of SSR markers used for QTL mapping were located in the genome sequence using a local BLAT server. Primers returning more than one potential amplification product were excluded and any primers amplifying products on scaffolds (un-anchored sections of the genome sequence that cannot currently be assigned to LGs) were excluded. For the purposes of extracting genes underlying QTL regions we produced R functions that first subset the genetic map to only those SSR markers that were located on the genome sequence. QTL regions were then defined by taking the flanking SSR markers from the location of the QTL and subsequently extracting a list of all genes between the genomic coordinates of the SSR markers. This approach typically led to extension of the QTL region beyond that of the QTL mapping confidence interval but occasionally led to a smaller region. We considered other approaches such as converting between cM and bp but such approaches are complicated by variable recombination frequencies both between and within linkage groups (data not shown).

Figure 2 was produced using Cytoscape [43]. Spearman's Rank correlation values were used as edge weights and trait names as nodes. The data matrix for use in Cytoscape was created using a custom R script and the igraph R package [44].

## Abbreviations

QTL: Quantitative Trait Loci; CC1: Coppice Cycle 1; CC2: Coppice Cycle 2; CC2-4: Coppice Cycle 2 Year 4; LG: Linkage Group; PBL: Poplar Biomass Loci; % Vp: percentage variance explained; SSR: Simple Sequence Repeat; SRC: Short Rotation Coppice; SS: Single Stem; CC1-1: Coppice Cycle 1 Year 1; CC: Coppice Cycle; WUE: Water Use Efficiency; PHYB2: Phytochrome B2; SNP: Single Nucleotide Polymorphism; CI: Confidence Interval.

## Authors' contributions

AMR performed and interpreted the QTL mapping, calculated the genetic correlations and helped draft the manuscript. NRS interpreted QTL mapping results, performed the correlation analysis, identified genes within QTL regions and drafted the manuscript. KMR designed the field trial, collected the field data and contributed to the interpretation of phenotypic trait data. NH collected the field data for the final biomass harvest. GT supervised the project.

## Supplementary Material

Additional file 1 **Table S1**. Details of all genetic correlations for all traits measured. Click here for file

Additional file 2 **Table S2**. Details of all QTL mapped. Click here for file
